# Supplementary material for: Bone morphogenetic protein 1.3 inhibition decreases scar formation and supports cardiomyocyte survival after myocardial infarction
Source: Nat Commun. 2022 Jan 10;13:81. doi: 10.1038/s41467-021-27622-9 (PMC8748453; doi:10.1038/s41467-021-27622-9)

Bone Morphogenetic Protein 1.3 inhibition decreases scar formation and supports cardiomyocyte survival after myocardial infarction

Slobodan Vukicevic[1#], Andrea Colliva[2,3#], Vera Kufner[1], Valentina Martinelli[4], Silvia Moimas[4], Simone Vodret[2], Viktorija Rumenovic[1], Milan Milosevic[5], Boris Brkljacic[6], Diana Delic-Brkljacic[7], Ricardo Correa[2], Mauro Giacca[3,4,8], Manuel Maglione[9], Tatjana Bordukalo-Niksic[1], Ivo Dumic-Cule [1#] and Serena Zacchigna[2,3#].

SUPPLEMENTARY INFORMATION

SUPPLEMENTARY FIGURES

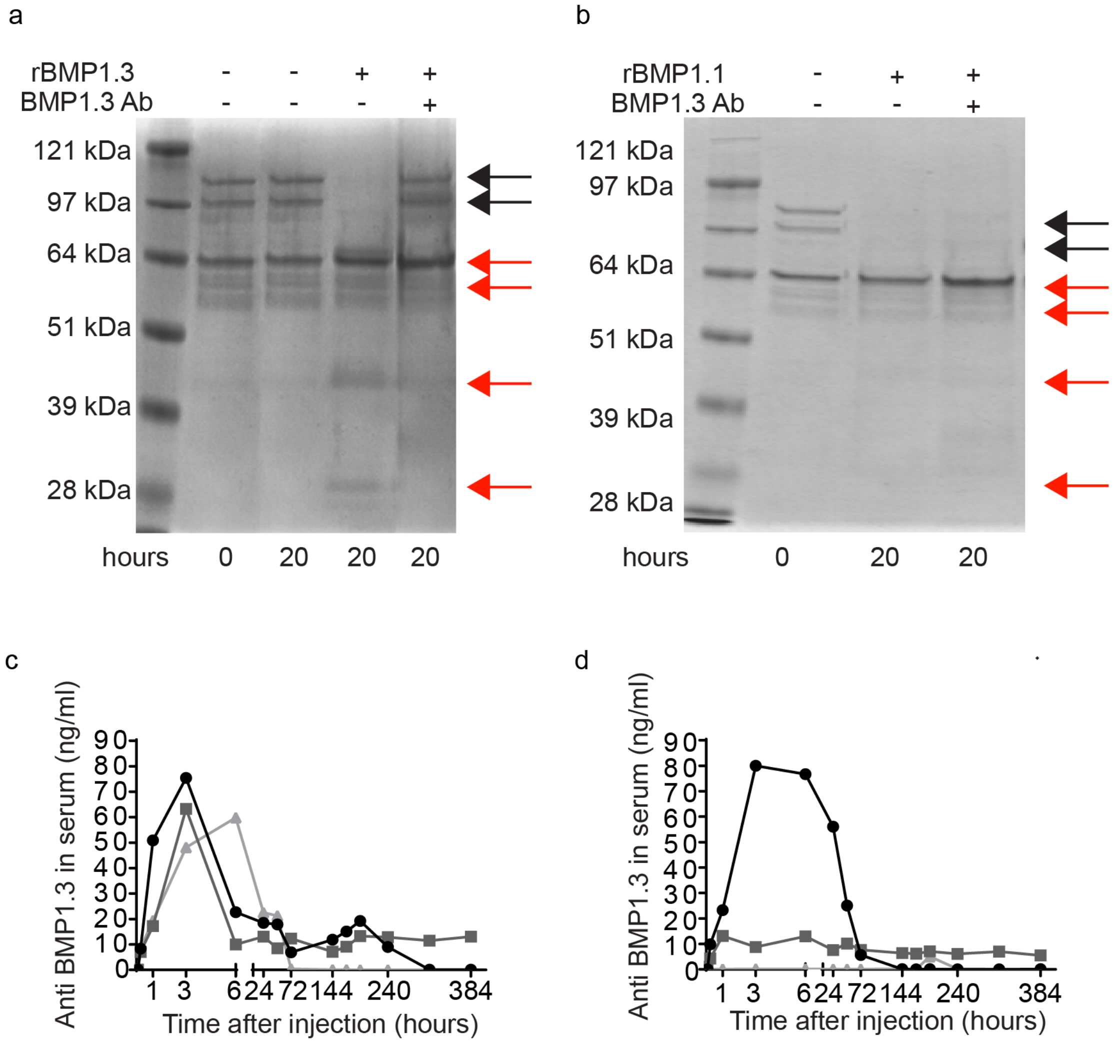

SUPPLEMENTARY FIGURE 1. Characterization of anti BMP1.3 antibody

a. Coomassie staining of DMP-1 full-length and its cleaved fragments incubated for the indicated time points with BMP1.3 recombinant protein, either alone or in combination with anti-BMP1.3 antibody. The first two lanes show DMP-1 stability between time 0 (immediate loading on gel) and after 20 hour-incubation in the same buffer. **b.** Coomassie staining of DMP-1 full-length and its cleaved fragments incubated for the indicated time points with BMP1.1 recombinant protein, either alone or in combination with anti-BMP1.3 antibody. In **a** and **b** experiments were repeated independently three times with similar results. **c, d.** Pharmacokinetic curve of the monoclonal mouse anti-BMP1.3 antibody, showing its concentration in the serum after a single dose intravenous (**c**) or intraperitoneal (**d**) injection in three rats over 384 hours. For details see Methods.

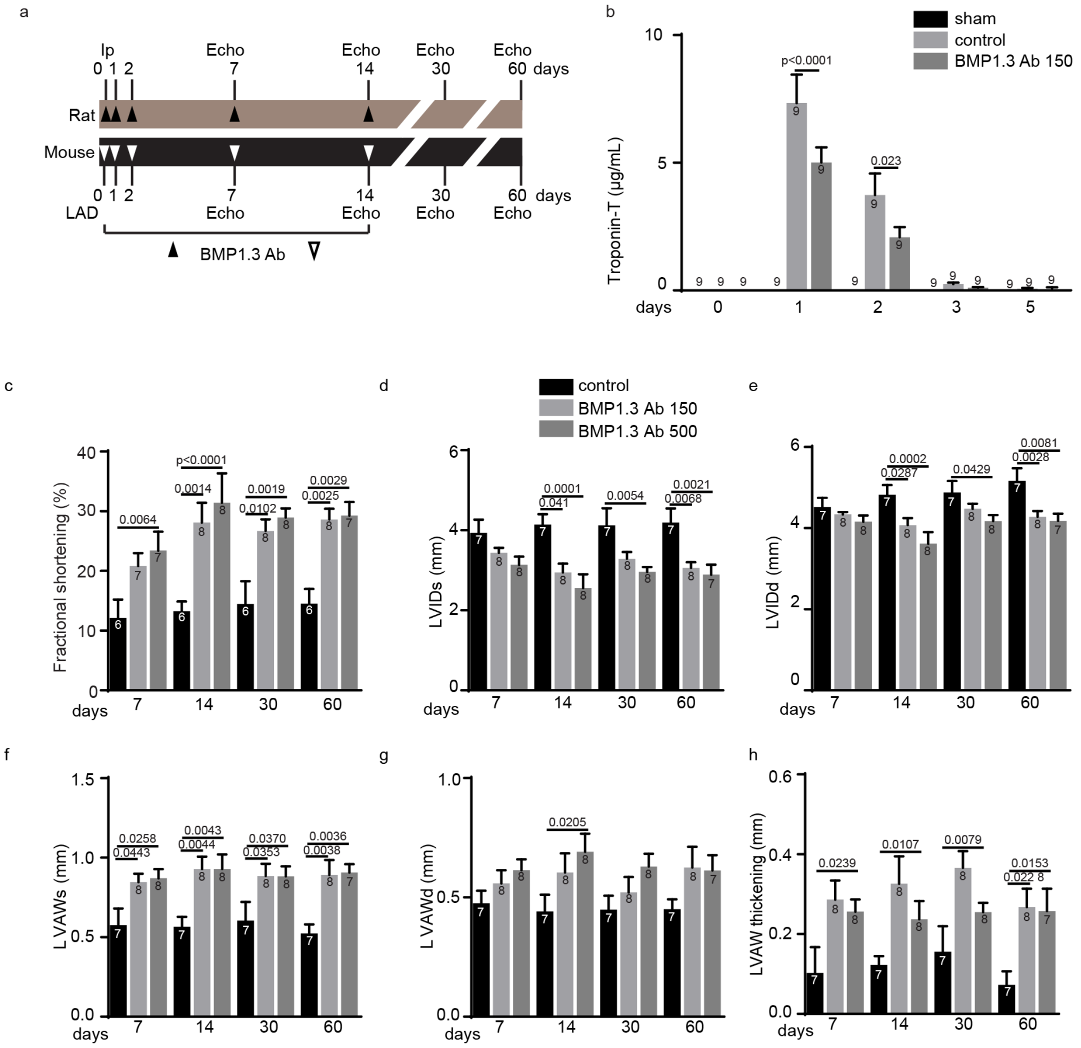

SUPPLEMENTARY FIGURE 2. Additional parameters of cardiac function in isoproterenol-treated rats and infarcted mice.

a. Schematic representation of anti-BMP1.3 antibody administration (arrowheads) and echocardiography in mice subjected to MI by left anterior descendent (LAD) coronary artery ligation (black timeline) and rats treated with isoproterenol (Ip, grey timeline). **b**. Quantification of Troponin-T levels in plasma of control rats (sham), rats treated with isoproterenol alone (control) or in combination with anti-BMP1.3 antibody (150 µg/kg) at the indicated time points. **c-h**. Quantification of Fractional shortening (**c**) end-systolic Left Ventricular Internal Diameter (**d**, LVIDs), end-diastolic Left Ventricular Internal Diameter (**e**, LVIDd), end-systolic Left Ventricular Anterior Wall thickness (**f**., LVAWs), end-diastolic Left Ventricular Anterior Wall thickness (**g**, LVAWd) and Left Ventricular Anterior Wall thickening (**h**) in infarcted mice in the absence of treatment (control) or treated with the indicated dose of anti-BMP1.3 antibody. Data in **b-h** are shown as mean±s.e.m. Sample number is indicated inside or above each bar. Statistical significance was determined using two-way ANOVA followed by Tukey's multiple comparison test in **b-h**. Source data are provided as a Source Data file.

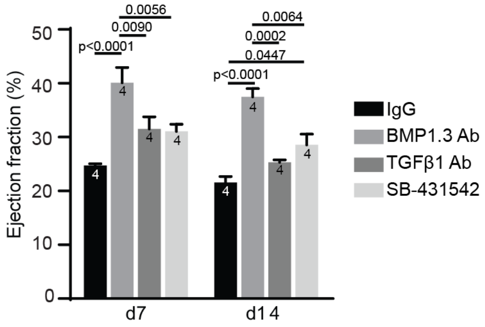

SUPPLEMENTARY FIGURE 3. Comparative analysis of cardiac function in mice treated with different anti-fibrotic drugs after MI.

Evaluation of the ejection fraction in mice subjected to MI and randomly assigned to the following experimental groups: i) IgG1 (isotype control); ii) anti-BMP1.3 antibody; iii) anti-TGFβ1 antibody, iv) SB-431542 (small molecule blocking multiple TGFβ receptors). Data are shown as mean±s.e.m. Sample number is indicated inside or above each bar. Statistical significance was determined using two-way ANOVA followed by Bonferroni's multiple comparison test. Source data are provided as a Source Data file.

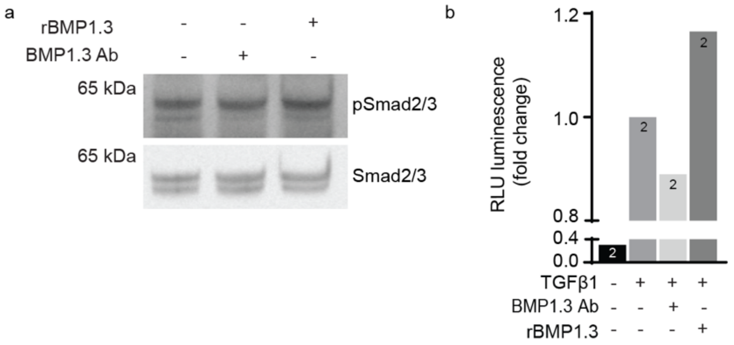

SUPPLEMENTARY FIGURE 4. Modulation of Smad2/3 phosphorylation by recombinant BMP1.3 and anti-BMP1.3 antibody

a. Western Blotting showing the level of total Smad2/3 and its phosphorylated form in primary murine fibroblasts treated with both recombinant BMP1.3 (0.1 μg/mL) and anti-BMP1.3 antibody 3 (5 μg/mL) for 48 hours. **b.** Luciferase assay on HEK293T stably expressing the TGFβ sensitive (CAGA)12-luciferase reporter, activated with TGFβ (2.5 pg/mL) for 17 hours, with either anti-BMP1.3 antibody 3 (5 μg/mL) or recombinant BMP1.3 (0.1 μg/mL). Data in **b** are shown as mean. Sample number is indicated above or in each bar. Source data are provided as a Source Data file.

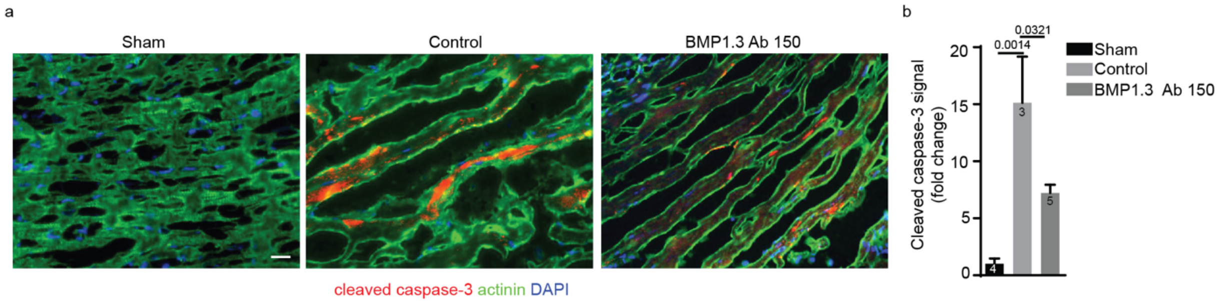

SUPPLEMENTARY FIGURE 5. Modulation of cardiomyocyte apoptosis by anti BMP1.3 antibody.

a. Representative images of cleaved caspase-3 and α-actinin staining on heart sections of mice untreated (sham) or injured by a myocardial infarction in the absence of treatment (control) or after injection of anti-BMP1.3 antibody (150 µg/kg). Nuclei were stained with DAPI. **b**. Quantification of apoptotic cells in the infarcted area. Scale bar indicates 10 µm. Data in **b** are shown as mean±s.e.m. Sample number is indicated inside or above each bar. Statistical significance was determined using one-way ANOVA followed by Tukey's multiple comparison test in **b**. Source data are provided as a Source Data file.

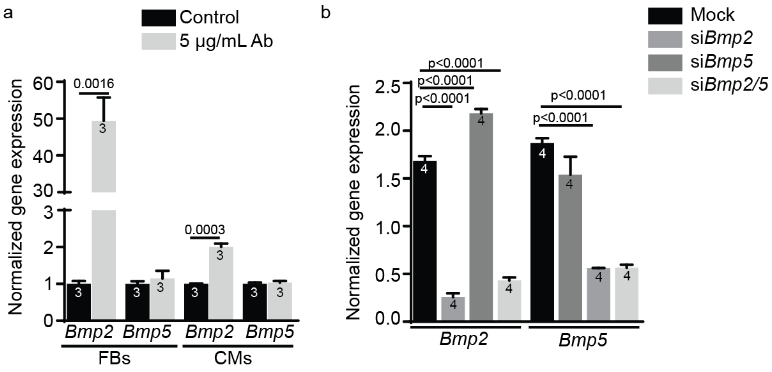

SUPPLEMENTARY FIGURE 6. Role of Bmp2 and Bmp5 in cardiomyocyte survival
a. Quantification of the expression levels of *Bmp2* and *Bmp5* in primary cardiac fibroblasts and cardiomyocytes in the absence of treatment (control) or treated with anti-BMP1.3 antibody (5 µg/ml), normalized to *Gapdh,* in normoxia. **b**. Quantification of the expression levels of *Bmp2* and *Bmp5* in primary cardiomyocytes transfected with the indicated siRNAs (mock = scramble siBMP5). Data shown as mean±s.e.m. Sample number is indicated inside or above each bar. Statistical significance was determined using unpaired, two-sided t-test in **a** or one-way ANOVA followed by Dunnet's multiple comparison test in **b**. Source data are provided as a Source Data file.

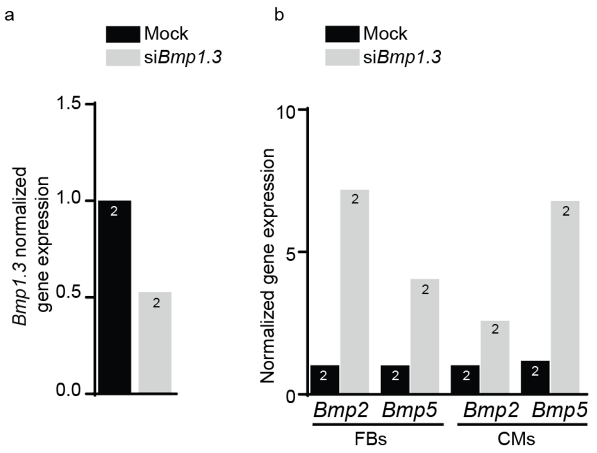

SUPPLEMENTARY FIGURE 7. Bmp1.3 silencing using siRNA recapitulates the effect of anti-BMP1.3 antibody

a. Quantification of the expression levels of *Bmp1.3* in primary fibroblasts transfected with siRNAs specific for BMP1.3 or a scramble sequence. **b.** Quantification of the expression levels of *Bmp2* and *Bmp5* in primary cardiac fibroblasts and cardiomyocytes treated with BMP1.3 specific siRNAs or a scramble sequence (mock), normalized to *Gapdh*. Data shown as mean. Sample number is indicated inside or above each bar. Source data are provided as a Source Data file.

Supplementary Methods

Validation of BMP1.3 ELISA kit

To validate our home-made ELISA, we followed the procedures indicated by Andreasson et al. 2015 (doi.org/10.3389/fneur.2015.00179).

First, we generated a standard curve using human recombinant BMP1.3 (8 biological replicates). As shown in Supplementary Figure 8, the assay provided reliable results in the 1,56 -100 ng/mL range. Shown are mean ± SD.

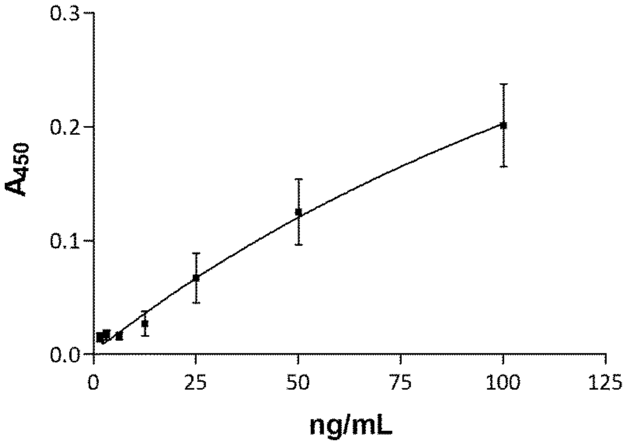

SUPPLEMENTARY FIGURE 8.

We then estimated the precision of the assay. First, we determined the following precision profile, based on back-calculated standard concentrations from 8 independent runs.

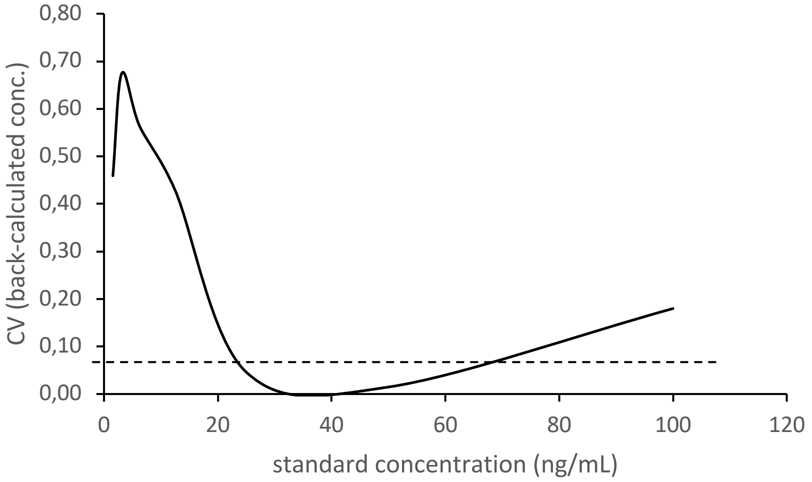

SUPPLEMENTARY FIGURE 9.

As evident from the profile curve in Supplementary Figure 9, the assay was precise in the 20-80 ng/mL range.

Additional precision experiments were performed on three different pooled samples divided in 15 aliquots. Five aliquots for each sample were analyzed in three independent runs in three different days. Results are shown in Supplementary Table 1.

	sample 1	sample 2	sample 3
mean concentration (ng/mL)	30,14	32,69	34,49
standard deviation	11,04	10,24	8,35
CV (%)	36,62	31,32	24,20

SUPPLEMENTARY TABLE 1.

To investigate if the concentration–response relationship was similar in the calibration curve and in samples, we performed a recovery test, by adding three different concentrations (5, 10 and 15 ng/sample) of recombinant BMP1.3 to human plasma samples and calculating the recovery using the following formula:

$$\% \text{ recovery} = \frac{(\text{observed conc. (spiked sample)} - \text{observed conc.(neat sample)})}{\text{expected conc.(spiked sample)}} \times 100$$

Supplementary Table 2 shows the recovery for 4 samples spiked with three different BMP1.3 concentrations.

	Spike level	Recovery (%)
sample 1	low	76,34
	medium	58,25
	high	43,71
sample 2	low	59,49
	medium	43,66
	high	41,69
sample 3	low	73,73
	medium	67,45
	high	42,62
sample 4	low	76,42
	medium	64,23
	high	45,38
sample 5	low	72,51
	medium	52,39
	high	50,46

SUPPLEMENTARY TABLE 2.

Supplementary Table 3. Sequences of primers used for gene expression analysis.

**Target gene**	**Forward 5′–3′**	**Reverse 5′–3′**
Rat *Bmp-2*	CCCTGAGTATCCCAATGGCTA	CCACATAGTCATACCAGCACAG
Rat *Bmp-4*	TGGGGAGGAGGAGGAAGAAG	CACTGGTCCCTGGGATGTTC
Rat *Bmp-5*	GACTTCGAGGCGGACACTTCTA	GCCGGTAAAGATCCCTCATGTAA
Rat *Bmp-6*	AGCAACAATCGCAACAGACG	GATCCAGCATGAAGAGCGGA
Rat *Bmp-7*	CCCTTCATGGTGGCCTTCTT	TTGGAGTCTTGGAGCGGTTC
Rat/Mouse *Ctgf*	CTGACCTAGAGGAAAACATT	AGAAAGCTCAAACTTGACA
Rat *Tgfb1*	GACTCTCCACCTGCAAGACC	GGACTGGCGAGCCTTAGTTT
Rat *Lox*	AAGGCACAGCGGACTTTCTC	GAACTCGTCCATGCTGTGGTAA
Rat *Col1a*	CACTGGCGATAGTGGTCCTG	CGGCCACCATCTTGAGACTT
Rat *Actb*	GCGCAAGTACTCTGTGTGGA	ACATCTGCTGGAAGGTGGAC
Mouse *Col1α1*	CGGCTCCTGCTCCTCTTAG	GGTTTCCACGTCTCACCATT
Mouse *Tgfb1*	GAAGGACCTGGGTTGGAAGT	CGGGTTGTGTTGGTTGTAGA
Mouse *Gapdh*	AGGTCGGTGTGAACGGATTTG	TGTAGACCATGTAGTTGAGGTCA
Mouse *Lox*	AAGGCACAGCGGACTTTCTC	GAACTCGTCCATGCTGTGGTAA
Mouse *Actb*	GCGCAAGTACTCTGTGTGGA	ACATCTGCTGGAAGGTGGAC
Rat/Mouse *Fn*	GACCACCACTCCCAAAAATG	TTGCAAACCTTCAATGGTCA
Mouse *Bmp1.3*	CCCTGAGTATCCCAATGGCTA	CCACATAGTCATACCAGCACAG
Human *BMP1.3*	ACAAGGACGAGTGCTCCAAG	GCGGCATTGGCACTCATAAC
Human *GAPDH*	CGGAGTCAACGGATTTGGTC	ATGAAGGGGTCATTGATGGCA

Supplementary information. Uncropped scans of blots in supplementary figure 4a. **a-c.** Uncropped pictures of the whole membrane (colorimetric picture, a) and the western blots from anti-Smad2/3 (Chemiluminescence, b) and anti-phosphorylated Smad2/3 (Chemiluminescence, c). Lanes 3, 4 and 5 correspond to those shown in supplementary figure 4a.

a

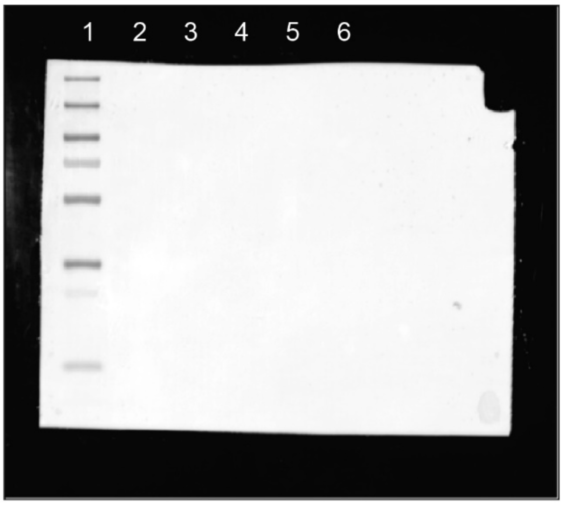

b

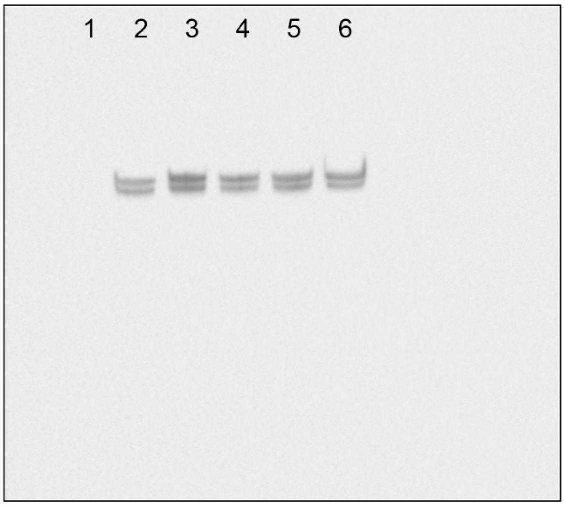

c

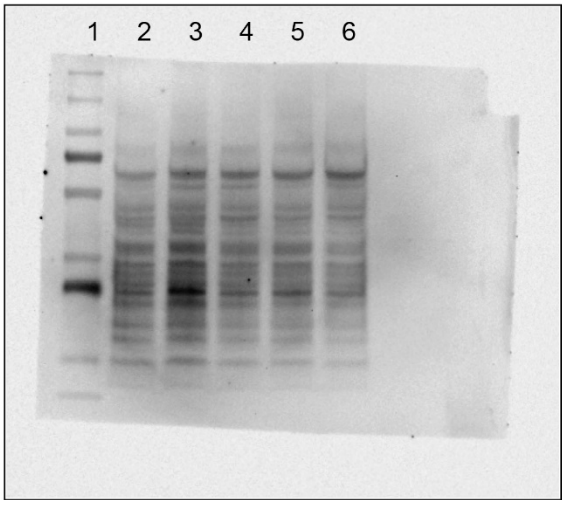

Supplement: Supplementary file 1 — Supplementary information [file 41467_2021_27622_MOESM1_ESM.pdf]
