# Supplementary material for: Bone morphogenetic protein 1.3 inhibition decreases scar formation and supports cardiomyocyte survival after myocardial infarction
Source: Nat Commun. 2022 Jan 10;13:81. doi: 10.1038/s41467-021-27622-9 (PMC8748453; doi:10.1038/s41467-021-27622-9)
Supplement: Supplementary file 3 — Reporting Summary [file 41467_2021_27622_MOESM3_ESM.pdf]

nature research

Corresponding author(s): Serena Zacchigna

Last updated by author(s): Oct 14, 2021

Reporting Summary

Nature Research wishes to improve the reproducibility of the work that we publish. This form provides structure for consistency and transparency in reporting. For further information on Nature Research policies, see our Editorial Policies and the Editorial Policy Checklist.

Statistics

For all statistical analyses, confirm that the following items are present in the figure legend, table legend, main text, or Methods section.

n/a	Confirmed	
☐	☒	The exact sample size (*n*) for each experimental group/condition, given as a discrete number and unit of measurement
☒	☐	A statement on whether measurements were taken from distinct samples or whether the same sample was measured repeatedly
☐	☒	The statistical test(s) used AND whether they are one- or two-sided<br>*Only common tests should be described solely by name; describe more complex techniques in the Methods section.*
☒	☐	A description of all covariates tested
☐	☒	A description of any assumptions or corrections, such as tests of normality and adjustment for multiple comparisons
☐	☒	A full description of the statistical parameters including central tendency (e.g. means) or other basic estimates (e.g. regression coefficient) AND variation (e.g. standard deviation) or associated estimates of uncertainty (e.g. confidence intervals)
☐	☒	For null hypothesis testing, the test statistic (e.g. *F*, *t*, *r*) with confidence intervals, effect sizes, degrees of freedom and *P* value noted<br>*Give P values as exact values whenever suitable.*
☒	☐	For Bayesian analysis, information on the choice of priors and Markov chain Monte Carlo settings
☒	☐	For hierarchical and complex designs, identification of the appropriate level for tests and full reporting of outcomes
☒	☐	Estimates of effect sizes (e.g. Cohen's *d*, Pearson's *r*), indicating how they were calculated

Our web collection on statistics for biologists contains articles on many of the points above.

Software and code

Policy information about availability of computer code

Data collection	Microscoft Excel Office 2019
Data analysis	Graphpad Prism 6, SPSS software (iBM SPSS Statistics version 2.2.0)

For manuscripts utilizing custom algorithms or software that are central to the research but not yet described in published literature, software must be made available to editors and reviewers. We strongly encourage code deposition in a community repository (e.g. GitHub). See the Nature Research guidelines for submitting code & software for further information.

Data

Policy information about availability of data

All manuscripts must include a data availability statement. This statement should provide the following information, where applicable:
- Accession codes, unique identifiers, or web links for publicly available datasets
- A list of figures that have associated raw data
- A description of any restrictions on data availability

Source data are provided with this paper

Field-specific reporting

Life sciences study design

All studies must disclose on these points even when the disclosure is negative.

Sample size	Sample size for animal experiments was determined using www.gpower.hhu.de to detect an effect with power = 0.8
Data exclusions	No data were excluded from the analyses of this work
Replication	All experiments in teh main figures have been successfully repeated at least three times.
Randomization	Animals involved in the same experiments were all syngenic and of the same age, so they have been randomly allocated in the different experimental groups
Blinding	All the processes of this work, from group allocation to data collection and analysis, have been performed in blind.

Reporting for specific materials, systems and methods

We require information from authors about some types of materials, experimental systems and methods used in many studies. Here, indicate whether each material, system or method listed is relevant to your study. If you are not sure if a list item applies to your research, read the appropriate section before selecting a response.

Materials & experimental systems

n/a	Involved in the study	
☐	☒	Antibodies
☐	☒	Eukaryotic cell lines
☒	☐	Palaeontology and archaeology
☐	☒	Animals and other organisms
☐	☒	Human research participants
☒	☐	Clinical data
☒	☐	Dual use research of concern

Methods

n/a	Involved in the study	
☒	☐	ChIP-seq
☒	☐	Flow cytometry
☒	☐	MRI-based neuroimaging

Antibodies

Antibodies used

- anti-BMP1.3 monoclonal antibody (clone No. 6B5A3) from Promab (Richmond, CA). in vitro neutralization assay: 5µg; in vivo administration: 50 µg/kg; in vitro administration: 5 µg/ml
- anti-BMP2 antibody (abcam ab6295) clone number: 65529.111. IHC: 1:200
- anti-BMP5 antibody (Thermo Fisher Scientific, PA5-78878). IHC: 1:1000
- anti-TGFβ1 antibody (Abcam, ab92486). IHC: 1:200
- anti-TGFβ1 antibody (BioXCell, BE0057). In vivo administration: 2 mg/kg
- anti-LOX2L (R&D Systems, MAB2639 clone number: 262418). IHC: 1:100
- anti-Sarcomeric Alpha Actinin antibody (Abcam, ab9465): IHC 1:3000 IF 1:200
- anti-SMAD2/3 (Cell Signalling Rabbit mAb #8685): WB: 1:1000
- anti-pSMAD2/3 antibodies (Cell Signaling Rabbit mAb #8828): WB: 1:1000
- anti-pSMAD2 (kind donation from Peter ten Dijke). IHC 1:100
- anti-cleaved Caspase 3 (Cell Signalling Rabbit pAb #9661). IF:1:100
- anti Donkey anti-Mouse IgG (H+L) Highly Cross-Adsorbed Secondary Antibody, Alexa Fluor 488 (Invitrogen A-21202). IF 1:500
- Mouse and Rabbit Specific HRP/AEC IHC Detection Kit - Micro-polymer; Abcam: ab236467
- goat anti-rabbit HRP-conjugated antibody (DAKO, ref p0448): WB: 1:2000

Validation

- anti-BMP1.3 monoclonal antibody (clone No. 6B5A3) from Promab (Richmond, CA). Monoclonal BMP1.3 antibody (internal code 84303#1; clone number 6B5A3) was produced by Promab Biotechnologies (Richmond, CA)) from specific peptide (sequence is included in the manuscript) upon order from the Laboratory of Mineralized Tissues, University of Zagreb School of Medicine, Zagreb, Croatia, and is not commercially available. Monoclonal BMP1.3 antibody was produced for laboratory use only.
- anti-BMP2 antibody (abcam ab6295) clone number: 65529.111. no validation statement available. datasheet at: https://www.abcam.com/bmp2-antibody-65529111-ab6285.html
- anti-BMP5 antibody (Thermo Fisher Scientific, PA5-78878). "no validation statement available". Testing data available at: https://www.thermofisher.com/antibody/product/BMP5-Antibody-Polyclonal/PA5-78878
- anti-TGFβ1 antibody (Abcam, ab92486). "Full length, inactive 44 kD TGFB1 is cleaved into mature TGFB1 (13 kD). TGFB1 also homodimerizes and heterodimerizes with TGFB2, so there is potential for multiple different band sizes in WB.ab92486 detects the mature secreted form of the protein at 13 kDa. Our internal testing data suggests that this does not detect the full length protein at 44kDa. As cell lysates are unsuitable for detection of the mature form, tissue lysates should be used when using this antibody." Testing data at <:https://www.abcam.com/tgf-beta-1-antibody-ab92486.html

anti-TGFβ1 antibody (BioXCell, BE0057). "Western Blot data confirm that this clone binds to its target antigen. For lot specific binding validation data, email technical service". Testing data at: https://bxcell.com/product/m-h-tgf-beta/
- anti-LOX2L (R&D Systems, MAB2639 clone number: 262418). no validation statement available. Datasheet at: https://

www.rndsystems.com/products/human-lysyl-oxidase-homolog-2-loxl2-antibody-262418_mab2639
- anti-Sarcomeric Alpha Actinin antibody (Abcam, ab9465): tested in WB: Mouse and rat skeletal muscle and rat heart tissue lysates. IHC-P: Human skeletal muscle tissue. Testing data at: https://www.abcam.com/sarcomeric-alpha-actinin-antibody-ea-53-ab9465.html
-anti-SMAD2/3 (Cell Signalling Rabbit mAb #8685):"This Ab has been validated using SimpleChiP Enzymatic Chromatin IP Kits". Testing data at: https://www.cellsignal.com/products/primary-antibodies/smad2-3-d7g7-xp-rabbit-mab/8685
-anti pSMAD (Cell Signaling Rabbit mAb # 8828): no validation statement available. Datasheet at: https://www.cellsignal.com/products/primary-antibodies/phospho-smad2-ser465-467-smad3-ser423-425-d27f4-rabbit-mab/8828
- anti-cleaved Caspase 3 (Cell Signalling Rabbit pAb : no validation statement available. Datasheet at: https://www.cellsignal.com/products/primary-antibodies/cleaved-caspase-3-asp175-antibody/9661
- anti Donkey anti-Mouse IgG (H+L) Highly Cross-Adsorbed Secondary Antibody, Alexa Fluor 488 (Invitrogen A-21202). no validation statement available. Testing data at: https://www.thermofisher.com/antibody/product/Donkey-anti-Mouse-IgG-H-L-Highly-Cross-Adsorbed-Secondary-Antibody-Polyclonal/A-21202
- Mouse and Rabbit Specific HRP/AEC IHC Detection Kit - Micro-polymer; Abcam: ab236467no validation statement available. Testing data at: https://www.abcam.com/mouse-and-rabbit-specific-hrpaec-ihc-detection-kit-micro-polymer-ab236467.html
- goat anti-rabbit HRP-conjugated antibody (DAKO, ref p0448): no validation statement available. datasheet at https://www.citeab.com/antibodies/3288347-p0448-goat-anti-rabbit-immunoglobulins-hrp-affinity

Eukaryotic cell lines

Policy information about cell lines

Cell line source(s)	HEK293T, Chinese Ovary Hamster CHO,
Authentication	Cell lines used in this study were not authenticated
Mycoplasma contamination	Not tested
Commonly misidentified lines (See ICLAC register)	None

Animals and other organisms

Policy information about studies involving animals; ARRIVE guidelines recommended for reporting animal research

Laboratory animals	Sprague-Dawley rats (age 2 days, both genders; 2 months , male); C57BL6 mice (age 2 months, male); aSMA-RFP/COLL-EGFP mice (age 2 months, male).
Wild animals	This study did not involve the use of wild animals
Field-collected samples	Thi study did not involve the use of sample collected by field
Ethics oversight	All animal experiments were conducted in accordance with guidelines from the Directive 2010/63/EU of the European Parliament on animal experimentation in compliance with European guidelines and International Laws and Policies (EC Council Directive 86/609, OJL 34, 12 December 1987) and were approved by the ICGEB Animal Welfare Board, the Ethical Committee and the Italian Ministry of Health (authorization number 1303/2015-PR) and by Institutional Animal Care Committee of Medical Faculty, University of Zagreb and Directorate for Veterinary and Food Safety, Ministry of Agriculture (Republic of Croatia), authorization number 525-10/0255-15-6.

Note that full information on the approval of the study protocol must also be provided in the manuscript.

Human research participants

Policy information about studies involving human research participants

Population characteristics	Patients of both genders, who suffered an acute myocardial infarction (AMI), and aged/gender matched healthy donors without comorbidities were recruited in this study. Among AMI patients, 6 had hyperlipidemia and others did not have significant comorbidities. All participants were older than 40 years.
Recruitment	Patients were consecutively recruited according to their admittance and controls were aged/gender matched. We do not foresee any major selection bias.
Ethics oversight	Study was approved by Ethical Committee of School of Medicine, University of Zagreb (EC KBC 5367001).

Note that full information on the approval of the study protocol must also be provided in the manuscript.
